# Reactive Behaviour of Platinum(II) Salts with Ethylenediamine in Sustainable Water/Choline Chloride-Based Deep Eutectic Solvents Mixtures

**DOI:** 10.3390/molecules30091890

**Published:** 2025-04-24

**Authors:** Nicola Garofalo, Francesco Messa, Alessandra Barbanente, Francesco Paolo Fanizzi, Antonio Salomone, Nicola Margiotta, Paride Papadia

**Affiliations:** 1 Department of Biological and Environmental Sciences and Technologies (DiSTeBA), University of Salento, 73100 Lecce, Italy; nicola.garofalo@unisalento.it (N.G.); francesco.messa@unisalento.it (F.M.); fp.fanizzi@unisalento.it (F.P.F.); 2 Dipartimento di Chimica, Università degli Studi di Bari Aldo Moro, Via E. Orabona 4, 70125 Bari, Italy; alessandrabarbanente@libero.it (A.B.); antonio.salomone@uniba.it (A.S.); nicola.margiotta@uniba.it (N.M.)

**Keywords:** deep eutectic solvents, cisplatin, platinum(II) complexes, DOSY, ^195^Pt NMR spectroscopy, ^15^N NMR spectroscopy, ^13^C NMR spectroscopy

## Abstract

Deep eutectic solvents (DESs) are environmentally friendly solvents formed by combining hydrogen bond donors and acceptors, resulting in a eutectic mixture with a lower melting point than the individual components. While there is extensive research on the electrochemical synthesis of platinum nanoparticles in DESs, to the best of our knowledge, there are no studies on the chemical reactivity of platinum(II) complexes in these systems. This study investigates the simple model reaction between K_2_PtCl_4_ and ethylenediamine (*en*), exploring the behaviour in DES environment, to optimize the synthesis of simple cisplatin-like platinum compounds with the potential objective of improving the traditional methods, decreasing the number of steps required for obtaining target compounds and reducing chemical waste. The reactions were performed in two hydrophilic DESs: choline chloride:glycerol 1:2 (glyceline, GL) and choline chloride:ethylene glycol 1:2 (ethaline, EG). The experiments, conducted in a 70% (*v*/*v*) DES and 30% 1:1 H_2_O/D_2_O mixture to allow for direct NMR analysis, revealed that *en* quickly formed [PtCl_2_(en)], which further reacted to produce [Pt(en)_2_]Cl_2_. Reaction products were characterised by 1D (^1^H and ^195^Pt{^1^H}) and 2D ([^1^H,^13^C]-HSQC and [^1^H,^15^N]-HSQC) NMR experiments. The discolouration of solutions, due to the consumption of K_2_PtCl_4_, and the precipitation of the purple Magnus salt [Pt(en)_2_][PtCl_4_] occurred over time. The main observed difference between the two solvent mixtures was the slower reactivity in glyceline, due to the much higher viscosity of the solution. Diffusion-ordered spectroscopy (DOSY) indicated lower water mobility in DES mixtures than pure water, with the reaction products closely associated with DES molecules.

## 1. Introduction

Deep Eutectic Solvents (DESs) are also known in the literature as Deep Eutectic Ionic Liquids (DEILs), Low Melting Mixtures (LMMs), or Low Transition Temperature Mixtures (LTTMs) [1]. They are usually obtained by combinations of two or more safe and inexpensive components which are a hydrogen bond acceptor (HBA), like halide salts, and a hydrogen bond donor (HBD) forming an eutectic mixture, that is a liquid at room temperature or slightly above, with a melting point lower than that of each individual component. The hydrogen bonding between, for example, a halide ion and the HBD moiety, is responsible for the decrease in the melting point of the mixture [2].

DESs share many characteristics with ionic liquids (ILs), including high thermal stability, low volatility, low vapour pressure, and tuneable polarity; however, ILs are usually expensive and nonbiodegradable, while DESs are typically inexpensive, biodegradable, non-toxic, and easier to prepare than ILs [3]. Furthermore, many ILs present some degree of toxicity [4]. In particular, the toxicity for the environment of imidazolium and pyridinium-based ILs is fully confirmed [5]. On the other hand, the natural and renewable origin of some components of DESs makes them promising candidates to replace toxic volatile organic solvents. Indeed, these eutectic mixtures have found use as green and bio-renewable solvents in many metal-catalysed organic reactions, like Pd-catalysed hydrogenation of organic compounds [6], Pd-catalysed aminocarbonylation of aryl iodide [7], or Pd-catalysed alkoxycarbonylation of aril iodides under gas-free conditions [8]. DESs were also applied as solvent to synthetize various nanostructured transition-metal materials [9].

While some attempts to investigate the chemical reactivity of platinum complexes was performed in ionic liquids [10,11,12], deep eutectic solvent have been mainly used as an environment for synthesising catalysts [13,14] and for metal extraction from different matrices [15]. Other examples of applications involve the selective separation and enrichment of platinum group metals (platinum, palladium, rhodium, iridium, ruthenium) [16], but to the best of our knowledge, no research is available on applications in the study of metal complexes’ synthesis and general reactivity. The lack of research on the subject prompted us to investigate the reactive behaviour of model compounds in green DESs. In fact, the typical synthetic procedure of anti-cancer platinum compounds in water solvents involves multiple steps, commonly including substitution of chloride ion with iodide, followed by removal of halogen ions through stoichiometric reaction with silver ions to afford precipitation of silver salts [17]. The non-traditional solvation behaviour and broad customisation of the reaction environment offered by DESs allow the development of tailored synthetic procedures so to avoid some of the aforementioned reaction steps. We therefore set out to study the synthesis of simple cisplatin-like platinum compounds (dichlorodiammine platinum) to assess the viability of certain low-footprint DESs for synthesis and explore the potential of the DES environment to streamline the synthesis process by reducing the number of steps required to obtain the target compounds, concurrently increasing the atom economy and reducing the need for disposal of chemical waste and/or recycling processes of metals with high energy costs. The hydrophilic DESs selected for the study were choline chloride:glycerol 1:2 (glyceline, GL) and choline chloride:ethylene glycol 1:2 (ethaline, EG).

## 2. Results

### 2.1. Behaviour and ^1^H NMR Spectroscopy of DES and Water Mixtures

**Figure 1:**
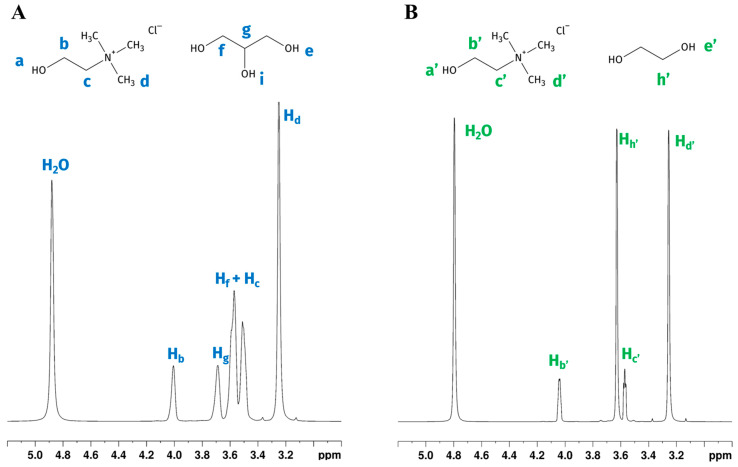
^1^H NMR spectra and chemical structures with atom labelling. (**A**) choline chloride-glycerol DES (GL); (**B**) choline chloride-ethylene glycol DES (EG). Lowercase blue (**A**) and green (**B**) letters near the structures were used to label the corresponding hydrogen signals in the spectra. Hydroxyl protons for both DESs are not detectable due to the chemical exchange with deuterated water.

NMR behaviour of choline chloride-glycerol (GL) and choline chloride-ethylene glycol (EG) DES mixtures added with various water percentages was previously studied. Water content affects the eutectic characteristics of DESs. Delso et al. [18] studied neat eutectic choline chloride (ChCl) based DESs and a mixture of DESs and different percentages of D_2_O (10% wt and 90% wt), detecting interactions among all species and finding that these ternary solvents still retain the eutectic character even when diluted at 10% wt. Ferreira et al. [19] studied ChCl/GLY 1:2 systems with added water (water contents ranging from 1 wt% to 70 wt%, using H_2_O and deuterated benzene as external standard), identifying three distinct water behaviour domains. Up to a water content of 11 wt%, water did not disrupt the DES structure; between water contents of 11 wt% and 35 wt%, the solvation of the DES components started to occur, but the DES nanostructure was still present. Hydrogen bond weakening was observed already at 5 wt% of water, while starting from 35 wt% of water, the DES structure itself is disrupted, with the system transitioning to a DES-in-water solution. Similar results on hydrogen bonding were reported for the choline chloride/ethylene glycol system by Gabriele et al. [20]. In our experimental conditions, the wt% of water was always lower than 28%; therefore it can be safely assumed that the solutions were in the intermediate case reported by Ferreira. ^1^H NMR spectra of GL and EG acquired with said D_2_O/H_2_O/DES mixture (1:1 H_2_O:D_2_O ratio) and the labelling of the corresponding DES components are reported in Figure 1. As previously reported by Ferreira, increasing the water content in mixtures causes a higher chemical exchange rate between water protons and hydroxyl of DESs [19]. Consequently, GL (H_a_, H_e_, and H_i_) and EG (H_a′_ and H_e′_) signals were not detectable in our mixtures, while a single signal belonging to water resonated at 4.88 and 4.79 ppm, respectively, in the spectra of GL:water and EG:water mixture (Figure 1A,B). The chemical shifts obtained for the two ternary DES solutions were very similar to those previously reported [18,19]. Signals were broad, not allowing us to appreciate multiplicities. Except for the overlapping signals belonging to H_c_ (methylene group bounded to the choline nitrogen) and H_f_ (methylenes of the glycerol), other signals were well separated, with the following assigned chemical shifts: 4.03 ppm (H_b_), 3.53 ppm (H_c_), and 3.24 ppm (H_d_) relative to choline, and 3.62–3.50 ppm (H_f_) and 3.74 ppm (H_g_) belonging to glycerol portion. A single water residual signal was found at δ = 4.88 ppm. Figure 1B shows the ^1^H NMR spectrum of EG, with the signals of choline assigned as follows: 4.04–4.03 ppm (H_b′_), 3.58–3.56 ppm (H_c′_), 3.25 ppm (H_d′_). The ethylene glycol shows a single signal at 3.62–3.66 ppm (H_h′_). The water protons resonate at 4.79 ppm.

### 2.2. Reactivity of K_2_PtCl_4_ with Ethylenediamine

Due to the lack of information about the reactivity of platinum(II) salts in DESs, we set out to investigate a simple model reaction between K_2_PtCl_4_ and ethylenediamine (*en*). The ethylenediamine bidentate ligand was preferred to perform the investigation, in place of two NH_3_ molecules (as those found in the clinically used antitumor drug cisplatin), to have readily available non-exchanging hydrogen atoms that could be tracked by ^1^H NMR spectroscopy.

**Scheme 1:**
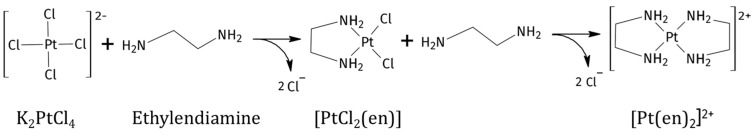
Reaction products of ethylenediamine to K_2_PtCl_4_ (K^+^ is omitted in the scheme for clarity).

The reaction between K_2_PtCl_4_ and *en* in traditional solvents can afford the products [PtCl_2_(en)] and [Pt(en)_2_]Cl_2_, depending on relative reaction speeds and stoichiometry of the ligand (Scheme 1) [21,22]. In this case, the reaction was performed in the two DES:water mixtures both at 1:1 and 1:2 Pt/en ratios. The reaction products were characterised by ^1^H, [^1^H,^13^C]-HSQC, [^1^H,^15^N]-HSQC, and ^195^Pt{^1^H} NMR spectroscopy.

### 2.3. Reactivity in Glyceline (Reaction 1)

The reaction between K_2_PtCl_4_ and *en* is normally performed under controlled conditions in water (low temperature and slow addition of reagents) [21] in order to obtain specifically the mono-addition product [PtCl_2_(en)] with a minimal amount of the double addition product [Pt(en)_2_]^2+^ as a side product; however in all DES:water solvents and under both stochiometric and *en* excess conditions, the reaction led to quick formation of [PtCl_2_(en)], followed by further reaction of an additional *en* moiety with the previously formed [PtCl_2_(en)] complex, affording [Pt(en)_2_]Cl_2_. Dissolved K_2_PtCl_4_ in both selected DESs made the solutions red-orange. As the reaction with *en* went on, K_2_PtCl_4_ was consumed, and we could observe discolouration of the solution, which turned transparent, the disappearance of [PtCl_2_(en)] peak in the spectra, and the unexpected precipitation of the previously described purple Magnus-type salt [Pt(en)_2_][PtCl_4_] [23].

#### 2.3.1. ^1^H and ^195^Pt NMR Experiments (Reaction 1)

**Figure 2:**
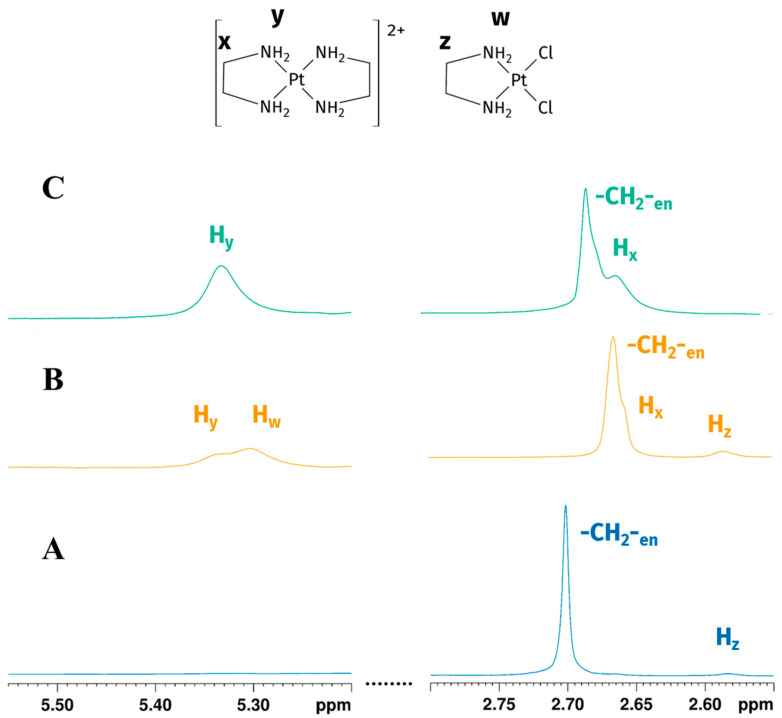
Expansions of the ^1^H NMR spectrum of Reaction 1 in the GL:water mixture 70:30% *v*/*v*: (**A**) -CH_2_-_en_ and H_z_ (-CH_2_- of [PtCl_2_(en)]) peaks at the beginning of the reaction show the early formation of mono-addiction product; (**B**) -CH_2_-_en_, H_z_, H_w_ (-CH_2_- and -NH_2_ of [PtCl_2_(en)]) and H_x_, H_y_ (-CH_2_- and -NH_2_ of [Pt(en)_2_]^2+^) peaks after four hours indicate the reaction goes on, reaching the formation of biscationic product; (**C**) -CH_2_-_en_ and H_x_, H_y_ (-CH_2_- and -NH_2_ of [Pt(en)_2_]^2+^) peaks 24 h later showing the consumption of [PtCl_2_(en)] (peaks are not to scale).

A 1:1 Pt-to-*en* reaction in GL:water 70:30% (*v*/*v*) mixture was performed under vigorous stirring conditions, obtaining a red-orange solution. ^1^H-NMR assignment of GL chemical shifts was performed according to the previously reported literature data [18,19]. Firstly, two peaks at 2.70 ppm and 2.58 ppm were observed (Figure 2A). Methylene signal of *en* in the reaction environment had the same chemical shift in ^1^H NMR spectrum of *en* in an EG:water 70:30% *v*/*v* solution, similar to the reported signal in water (at 2.65 ppm) [24]; methylene signals belonging to *en* following coordination to the metal centre resonated upfield, giving a shielded signal at 2.58 ppm [21]. Exchangeable -NH_2_ protons of *en* were not detected, possibly due to fast chemical exchange with deuterated water.

Four hours later we observed a partial consumption of *en* and the presence of new peaks, respectively, at 2.66, 5.31, and 5.34 ppm (Figure 2B). The two deshielded peaks on the left of the water signal (5.31 and 5.34 ppm) are characteristic of -NH_2_ coordinated with platinum(II) [25]. To further confirm the coordination of *en,* ^195^Pt-NMR experiments were performed.

**Figure 3:**
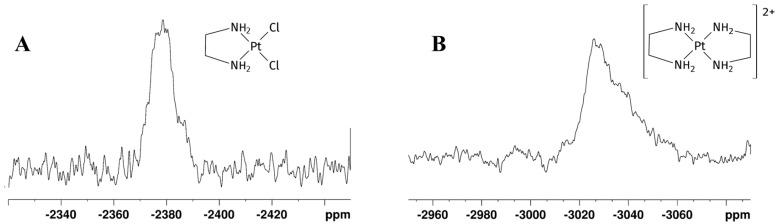
^195^Pt NMR spectra recorded after 24 h on the mother solution of the reaction reported in Scheme 1 performed in GL:water 70:30% *v*/*v* mixture showing (**A**) [PtCl_2_(en)] and (**B**) [Pt(en)_2_]^2+^ peaks. The detected δ(^195^Pt) [PtCl_2_(en)] = −2384 ppm, δ(^195^Pt) [Pt(en)_2_]^2+^ = −3030 ppm.

^195^Pt chemical shielding has a wide range of values, depending on the oxidation state of the metal, on the nature of all coordinated ligands (element and oxidation state) and, to a minor extent, on the solvation. In particular, for a given oxidation state, the ^195^Pt chemical shift is highly diagnostic of the molecular structure of the complex, with each ligand giving a predictable contribution to the value of the complex chemical shift [26,27]. δ_Pt_ for Pt(II) complexes change according with spectroscopical ordering of ^195^Pt shielding Cl > NO_2_ > NH_3_ > Br > *en* > SCN > CN > I [26]. In GL:water 70:30% (*v*/*v*) K_2_PtCl_4_ showed a peak at −1560 ppm, which appeared slightly deshielded compared to that observed in D_2_O (−1617 ppm) [28]. This deshielding effect is smaller compared to the differences in chemical shifts reported for various other organic solvents [27]. The substitution of two chloride ligands by one *en* molecule gave rise to [PtCl_2_(en)], whose signal appears more shielded than the signal belonging to K_2_PtCl_4_ and was observed at −2384 ppm (Figure 3A), in agreement with a PtN_2_Cl_2_ coordination sphere. Due to the low solubility of [PtCl_2_(en)] in water, the chemical shifts could not be directly compared; nevertheless, this species was reported at −2345 ppm in DMSO-d_6_ [29]. This allowed us to assign the protons belonging to -CH_2_-(H_z_) and -NH_2_ (H_w_) of the mono-addition product at 2.58 and 5.31 ppm, respectively. After 24 h, the ^1^H spectrum showed the disappearance of the [PtCl_2_(en)] peak, a residual free -CH_2_- *en* peak at 2.67 ppm, and two unassigned peaks at 2.66 and 5.34 ppm (Figure 2C). Full ^1^H NMR stacked spectra for the reaction are reported in Figure S1 in Supplementary Materials.

A further ^195^Pt experiment showed a new shielded signal at −3030 ppm (Figure 3B), typical of a PtN_4_ coordination sphere, confirming coordination of another *en* moiety to [PtCl_2_(en)], affording [Pt(en)_2_]Cl_2_. The chemical shift of [Pt(en)_2_]Cl_2_ was previously reported at −3015 ppm in D_2_O [26].

This allowed us to confirm the assignment of the two peaks at 2.66 and 5.34 in the ^1^H-NMR spectrum, respectively, to -CH_2_- (H_x_) and -NH_2_ (H_y_) of bis-cationic product.

Concurrently, the discolouration of the solution was observed, along with the precipitation of a purple Magnus-type salt ([Pt(en)_2_][PtCl_4_]), in agreement with the consumption of the coloured K_2_PtCl_4_ species, as further confirmed by the disappearance of the corresponding peak in the ^195^Pt spectrum [23,30].

#### 2.3.2. [^1^H,^13^C]-HSQC and [^1^H,^15^N]-HSQC NMR Experiments (Reaction 1)

**Figure 4:**
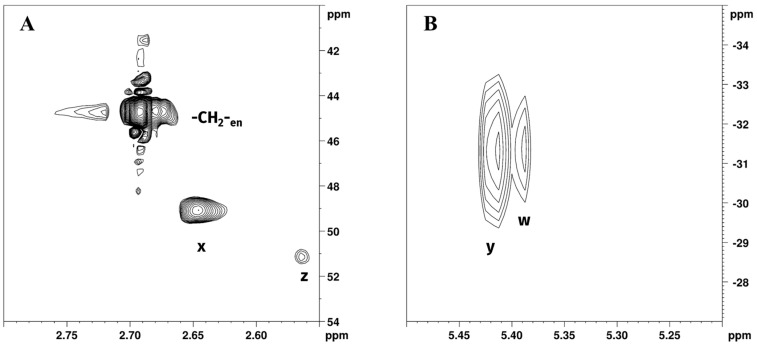
Expansions of (**A**) [^1^H,^13^C]-HSQC and (**B**) [^1^H,^15^N]-HSQC NMR spectra of Reaction 1 in GL:water 70:30% *v*/*v* mixture showing -CH_2_-_en_; z, w (-CH_2_- and -NH_2_ of [PtCl_2_(en)]) and x, y (-CH_2_- and -NH_2_ of [Pt(en)_2_]^2+^) peaks.

[^1^H,^13^C]-HSQC and [^1^H,^15^N]-HSQC NMR spectra (Figure 4), acquired in the following 4 h, were used to further characterise reaction products, and data are reported in Table 1.

Chemical shift assignments were made on the basis of previously obtained results from the ^1^H and ^195^Pt spectra. Expansion of [^1^H,^13^C]-HSQC NMR spectrum of Reaction 1 in GL:water solution 70:30% *v*/*v* (Figure 4A) showed cross peaks at 2.68/45.90 ppm, belonging to -CH_2_- of free *en*, 2.65/49.14, and 2.57/51.21 ppm corresponding to [Pt(en)_2_]^2+^ and [PtCl_2_(en)], respectively. This allowed us to resolve the partially overlapping ^1^H NMR signals of -CH_2_-_en_ and H_x_ (-CH_2_- of [Pt(en)_2_]^2+^), reported in Figure 2B. Figure 4B shows the expansion of the [^1^H,^15^N]-HSQC NMR spectrum for Reaction 1 in GL:water solution 70:30% *v*/*v*. As previously noted, the coordination of amine to Pt(II) results in a characteristic upfield shift of ^15^N signals compared to free amines; moreover, the degree of shielding shows a characteristic dependence on ligand trans to the nitrogen atom [31]. Peaks at 5.39/−31.37 and 5.41/−31.42, were assigned to [PtCl_2_(en)] and [Pt(en)_2_]^2+^, respectively. These data are consistent with previously reported ^15^N chemical shift of ethylenediamine coordinated to Pt, in which -NH_2_ signals *trans* to N are found at −33.0 and −30.5 ppm [32]. Exchangeable -NH_2_ protons belonging to free *en* were not detected in [^1^H,^15^N]-HSQC NMR, possibly due to fast chemical exchange with deuterated water.

Following the consumption of K_2_PtCl_4_ through the formation of the Magnus-type salt, free ethylenediamine and [Pt(en)_2_]Cl_2_ could be detected in the solution for 30 days at 7 °C through ^1^H and [^1^H,^13^C]-HSQC NMR (Figure S3A).

The reaction between K_2_PtCl_4_ and *en* was repeated using a 1:2 ratio between the reagents: no significant differences were observed in the appearance of the products or the consumption of K_2_PtCl_4_. Discolouration of the solution was observed after 24 h also under these experimental conditions.

#### 2.3.3. DOSY and T1 Experiments (Reaction 1)

**Figure 5:**
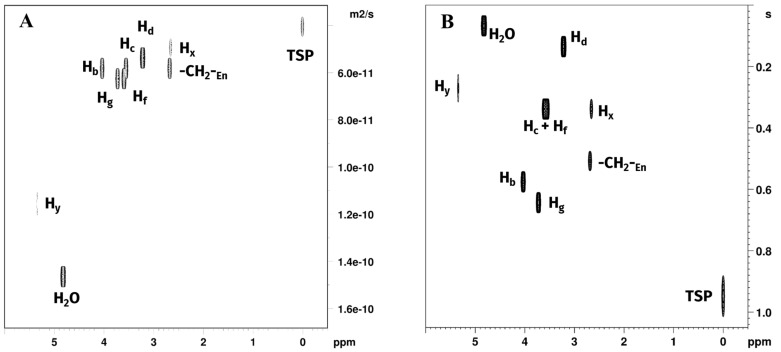
Plots of (**A**) DOSY and (**B**) T1 relaxation time experiments of Reaction 1 in GL:water 70:30% (*v*/*v*) mixture showing signals belonging to water, GL, TSP, free *en* and [Pt(en)_2_]^2+^. [PtCl_2_(en)] was not observed due to rapid consumption in the reaction environment.

Diffusion ordered spectroscopy (DOSY) experiments, measuring the diffusion coefficient D, and inversion recovery experiments, measuring spin–lattice relaxation time T1, were performed to obtain information about, respectively, molecular mobility and interactions with the solvation environment of choline chloride, glycerol, water, free *en*, and reaction products (Figure 5A,B). Corresponding fitted diffusion coefficients and T1 values are reported in Table 2.

Since [PtCl_2_(en)] was rapidly consumed in the reaction environment, a separately synthesized [PtCl_2_(en)] standard was added to the GL:water mixture 70:30% *v*/*v* to measure its diffusion coefficient and relaxation time in the mixture, following reaction completion.

Diffusion coefficients measured for choline chloride and glycerol hydrogen atoms were all close to each other (~0.5 × 10^−10^ m^2^/s), with slightly higher values for glycerol H_f_ and H_g_ (~0.55 × 10^−10^ m^2^/s), indicating a marginally higher mobility of glycerol in the solution. Water molecules diffusion coefficient was more than twice greater than those measured for the DES components, indicating comparatively higher mobility of the water in the mixture, while still being more than one order of magnitude smaller than the one observed in pure water (D_H2O_ = 2.19 × 10^−9^ m^2^·s^−1^) [33]. The non-labile CH_2_ protons of *en* showed diffusion coefficients closer to those measured for the DES components, with the non-coordinated *en* being the fastest moving (D = 0.52 × 10^−10^ m^2^/s), with a diffusion coefficient greater than choline chloride but smaller than glycerol, followed in order by [PtCl_2_(en)] (H_z_) and [Pt(en)_2_]^2+^ (H_x_). Both the complexes showed a lower D than any component of the DES. The -NH_2_ protons of [PtCl_2_(en)] (H_w_) and [Pt(en)_2_]^2+^ (H_y_) both showed a D higher than 1.0 × 10^−10^ m^2^/s, due to significant exchange with water.

T1 values are the time constants measuring the speed of the process by which nuclear spins return to thermal equilibrium with their surrounding environment following excitation by an external radiofrequency pulse [34]. T1 values are in general correlated with the temperature of the sample, the external applied magnetic field, and the speed of molecular motion (molecular tumbling) in a non-linear fashion. Moreover, in a specific sample, T1 values for different nuclei in different molecules depend on their specific dipolar interactions, due to the solvation environment and the molecular environment. In the case of choline, the observed order of T1 was H_d_ < H_c_ < H_b_, with the fastest relaxation observed for the methyl and methylene groups bound to the quadrupolar nitrogen atom and in order of the amount of steric interaction. The slowest relaxation was observed for the CH_2_OH non-labile protons, which are expected to be the less mobile part of the molecule, being involved in the hydrogen bonding of the DES. In the case of glycerol, the terminal CH_2_OH H_f_ showed a faster relaxation than the H_g_. Ethylenediamine CH_2_ T1 were in the following order: H_z_ of [PtCl_2_(en)] < H_x_ of [Pt(en)_2_]^2+^ < free *en*. This appears consistent with the coordination to platinum, and with a molecular environment of [Pt(en)Cl_2_] more exposed to dipolar interactions. T1 value for H_y_ (-NH_2_ of [Pt(en)_2_]^2+^) was slightly faster than the value obtained for H_x_. H_w_ (-NH_2_ of [PtCl_2_(en)]) fitting was not possible due to a very low signal-to-noise ratio.

### 2.4. Reactivity in Ethaline (Reaction 2)

#### 2.4.1. ^1^H and ^195^Pt NMR Experiments (Reaction 2)

As in the previous case, 1:1 and 1:2 Pt-to-*en* reactions in EG:water 70:30% (*v*/*v*) mixture were performed under vigorous stirring condition, obtaining a red-orange solution. ^1^H-NMR assignment of EG chemical shifts, were in good agreement with previous reports in the literature [15].

**Figure 6:**
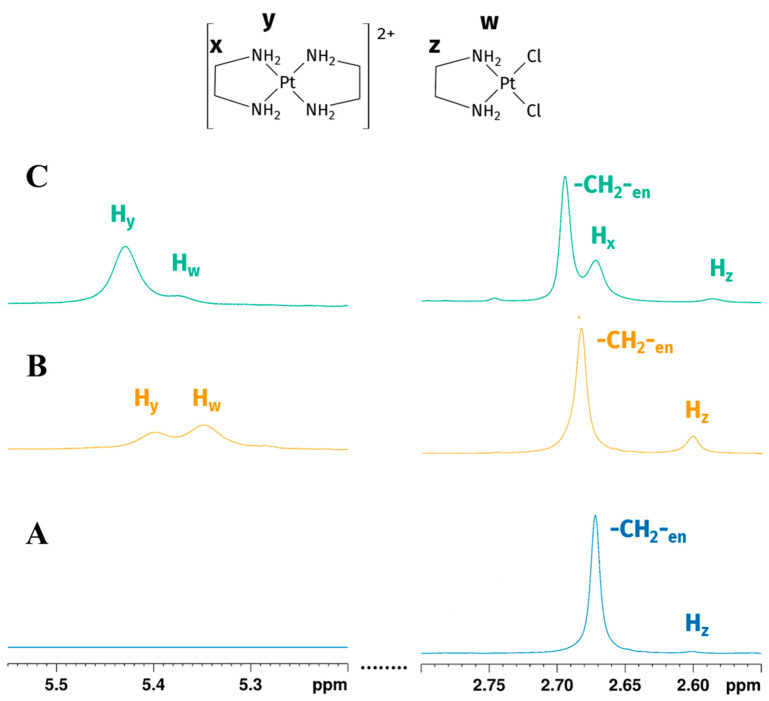
Expansions of the ^1^H NMR spectrum of Reaction 2 in the EG:water mixture 70:30% *v*/*v*: (**A**) -CH_2_-_en_ and H_z_ (-CH_2_- of [PtCl_2_(en)]) peaks at the beginning of the reaction show the early formation of mono-addiction product; (**B**) -CH2-en, H_z_, H_w_ (-CH_2_- and -NH_2_ of [PtCl_2_(en)]) and H_y_ (-NH_2_ of [Pt(en)_2_]^2+^) peaks after one hour (H_x_ signals overlaps with -CH_2_-_en_) indicate the reaction goes on, reaching the formation of biscationic product; (**C**) -CH_2_-_en_, H_z_, H_w_ (-CH_2_- and -NH_2_ of [PtCl_2_(en)]) and H_x_, H_y_ (-CH_2_- and -NH_2_ of [Pt(en)_2_]^2+^) peaks, acquired 24 h later, show the partial consumption of [PtCl_2_(en)] (peaks are not to scale).

For both Pt-to-*en* ratios, initially signals belonging to -CH_2_- of free *en* at 2.67 ppm were observed, followed by the appearance H_z_ (-CH_2_-) of [PtCl_2_(en)] at 2.60 ppm (Figure 6A). Exchangeable -NH_2_ protons belonging to *en* were not detected by ^1^H NMR. Methylene signal of *en* in the reaction environment showed the same chemical shift in ^1^H NMR spectrum of *en* in an EG:water 70:30% *v*/*v* solution. ^1^H NMR spectrum acquired 1 h later still showed -CH_2_- of free *en* at 2.68 ppm and H_z_ (-CH_2_-) of [PtCl_2_(en)] at 2.60 ppm. Moreover H_w_ (-NH_2_) of [PtCl_2_(en)] and H_y_ (-NH_2_) of [Pt(en)_2_]Cl_2_ started to be detectable at 5.35 ppm and 5.42 ppm, respectively (Figure 6B). The signal of H_x_ (-CH_2_-) of [Pt(en)_2_]Cl_2_ could not be singled out, due to overlapping with the free *en* signal. Twenty-four hours later, as the consumption of *en* continued, H_x_ and H_y_ signals of [Pt(en)_2_]Cl_2_ were observed, respectively, at 2.68 and 5.42 ppm, while -CH_2_- of free *en*, H_z_ and H_w_ could still be detected in the solution at 2.69, 2.60, and 5.35 ppm, respectively (Figure 6C). Full ^1^H NMR stacked spectra for the reaction are reported in Figure S2 in Supplementary Materials.

Also in this case, for both reagent ratios, we observed the precipitation of Magnus-type salt and the nearly total discolouration of the solution within 24 h. These changes resulted faster than those observed in the GL:water solution.

**Figure 7:**
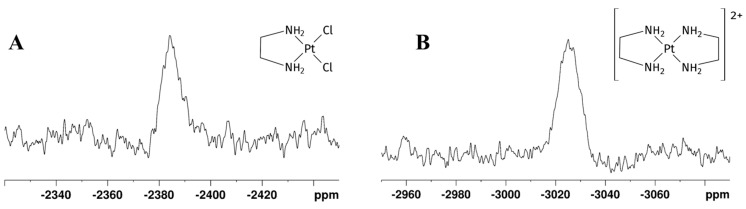
^195^Pt NMR spectra of Reaction 1 in EG:water 70:30% *v*/*v* mixture showing (**A**) [Pt(en)_2_]^2+^ and (**B**) [PtCl_2_(en)] peaks. δ(^195^Pt) for [PtCl_2_(en)] = −2386 ppm, [Pt(en)_2_]Cl_2_ = −3028 ppm.

In the ^195^Pt NMR spectra, the K_2_PtCl_4_ signal was observed at −1561 ppm, and the [PtCl_2_(en)] signal at −2380 ppm. As previously mentioned for Reaction 1, also in Reaction 2 we observed the shielding of [PtCl_2_(en)] signal at −2380 ppm, following the substitution of two chlorido ligands with one *en* molecule. The negligible difference with the chemical shifts observed in glyceline suggests a roughly comparable solvation environment for the complexes in the two ternary mixtures. Although [Pt(en)_2_]Cl_2_ signals were detected in ^1^H spectrum acquired after 1 h, they were not detected early in the ^195^Pt-NMR spectrum. ^195^Pt-NMR spectrum acquired 48 h later confirmed the complete K_2_PtCl_4_ and [PtCl_2_(en)] consumption and the coordination of another *en* moiety to metal centre in order to obtain [Pt(en)_2_]Cl_2_, whose signal appeared furtherly shielded at −3028 ppm. Peaks for the two products are reported in Figure 7.

#### 2.4.2. [^1^H,^13^C]-HSQC and [^1^H,^15^N]-HSQC NMR Experiments (Reaction 2)

**Figure 8:**
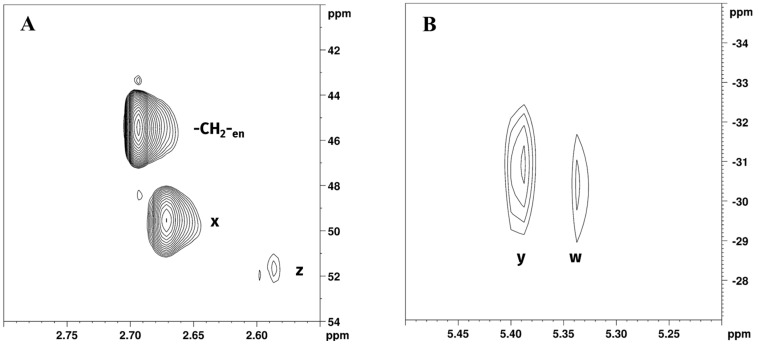
Expansions of (**A**) [^1^H,^13^C]-HSQC and (**B**) [^1^H,^15^N]-HSQC NMR spectra of Reaction 2 in EG:water 70:30% *v*/*v* mixture showing -CH_2_-_en_; z, w (-CH_2_- and -NH_2_ of [PtCl_2_(en)]) and x, y (-CH_2_- and -NH_2_ of [Pt(en)_2_]^2+^) peaks.

[^1^H,^13^C]-HSQC and [^1^H,^15^N]-HSQC NMR (Figure 8) spectra were used to further characterize reaction products. All chemical shift assignments are summarised in Table 3. Chemical shifts assignments were made on the basis of previously obtained results from the ^1^H and ^195^Pt spectra and resulted similar to those obtained from Reaction 1 in GL:water 70:30% *v*/*v* mixture.

Expansion of [^1^H,^13^C]-HSQC NMR spectrum of Reaction 2 in EG:water solution 70:30% *v*/*v* (Figure 8A) showed cross peaks at 2.69/45.44 ppm, belonging to -CH_2_- of free *en* (further confirmed by [^1^H,^13^C]-HSQC NMR of *en* in EG:water mixture 70:30% *v*/*v*, found at 2.67/45.36 ppm), 2.67/49.53, and 2.58/51.76 corresponding to [Pt(en)_2_]^2+^ and [PtCl_2_(en)], respectively. Expansion of the [^1^H,^15^N]-HSQC NMR spectrum of Reaction 2 in EG:water solution 70:30% *v*/*v* is reported in Figure 8B. Peaks at 5.34/−30.44 and 5.40/−30.93 corresponded to [PtCl_2_(en)] and [Pt(en)_2_]^2+^, respectively. Exchangeable -NH_2_ protons belonging to *en* were not detected in [^1^H,^15^N]-HSQC NMR.

Free ethylenediamine and [Pt(en)_2_]Cl_2_ could be detected in the solution 30 days later at 7 °C through ^1^H and [^1^H,^13^C]-HSQC NMR, while [PtCl_2_(en)] resulted completely consumed (Figure S3B).

#### 2.4.3. DOSY and T1 Experiments (Reaction 2)

**Figure 9:**
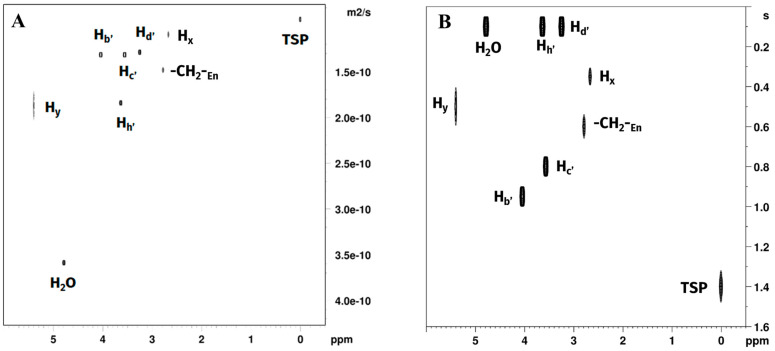
Plots of (**A**) DOSY and (**B**) T1 relaxation time experiments on the reaction products in EG:water 70:30 % *v*/*v* mixtures showing signals belonging to water, EG, TSP, free *en* and [Pt(en)_2_]^2+^. [PtCl_2_(en)] was not observed due to rapid consumption in the reaction environment.

DOSY experiments measuring mobilities and inversion recovery experiments measuring T1 relaxation times of the solution environment after reaction completion are reported in Figure 9A,B. Fitted diffusion coefficients and T1 values are reported in Table 4.

Diffusion coefficients for the choline chloride non-labile hydrogens were (~1.25 × 10^−10^ m^2^/s) significantly smaller than those observed ethylene glycol H_h′_ (1.78 × 10^−10^ m^2^/s), indicating a relatively higher mobility of the polyol in the solution. Water molecules diffusion coefficient was approximately twice as high as the one of DES components, indicating a relatively high motion of the water in the mixture (D = 3.57 × 10^−10^ m^2^·s^−1^), although still not comparable to the values observed in pure water (D_H2O_ = 2.19 × 10^−9^ m^2^·s^−1^) [33]. The non-labile CH_2_ protons of *en* coordinated to platinum showed diffusion coefficients smaller than those measured for any of the DES components (~1.0 × 10^−10^ m^2^/s, with [PtCl_2_(en)] (H_x_) > [Pt(en)_2_]^2+^ (H_z_)), while the non-coordinated *en* appeared to be faster (D = 1.41 × 10^−10^ m^2^/s) than choline chloride but slower than ethylene glycol. Diffusion coefficient values for the exchangeable -NH_2_ protons were both higher than those observed for the non-labile protons of the same species but concurrently lower than the one measured for water, indicating a slower exchange rate and possibly a higher interaction of the complex with the components of the DES. This seems to be the case particularly for [Pt(en)_2_]^2+^ (H_y_), having D_Hy_ = 1.78 × 10^−10^ m^2^/s, a value compatible with the measured mobility of ethylene glycol.

T1 values for choline were observed in the order H_d′_ < H_c′_ < H_b′_, with the fastest relaxation observed for the methyl and methylene groups bound to the quadrupolar nitrogen atom and in order of amount of steric interaction. The slowest relaxation time was observed for the -CH_2_OH non-labile protons, expected to be the less mobile part of the molecule, being involved in the hydrogen bonding of the DES. Moreover, the methyl groups have a significantly shorter T1 than the rest of the atoms of choline, suggesting an environment more susceptible to dipolar interaction. Even the terminal -CH_2_OH H_h′_ showed a very short T1, comparable to the one observed for water in this system. This seems to indicate a solution divided in two environments, one shared by ethylene glycol, water, and the methyl groups of choline, and another one allowing less dipolar interactions, containing the hydroxyethyl group of choline. Moreover, in the EG:water system ethylenediamine CH_2_ T1 observed values were in the order H_x_ < H_z_ < free *en*. This appears consistent with the coordination to platinum, and with the more crowded molecular environment of [Pt(en)_2_]Cl_2_. Interestingly, the T1 values of the complexes did not show changes of one order of magnitude as those shown by choline, suggesting minor adjustments in the solvation environments. The effect observed, however, appears to be more significant on [PtCl_2_(en)]. Considering that all molecules are of small size and can be expected to be affected similarly by changes in viscosity of the solution, this effect could be attributed to specific solvation changes for the neutral species. T1 values for H_y_ (-NH_2_ of [Pt(en)_2_]^2+^) were smaller than those obtained for H_x_. We were not able to detect H_w_ (-NH_2_ of [PtCl_2_(en)]) due to low signal-to-noise ratio.

### 2.5. Comparison of the Hydrogen Bond Donors Glycerol and Ehtylene Glycol

In both DES:water mixtures, and for both stochiometric and *en* excess conditions, the reaction led to the rapid formation of [PtCl_2_(en)], followed a few hours later by further reaction of an additional *en* moiety with the previously formed [PtCl_2_(en)] complex, affording [Pt(en)_2_]Cl_2_. In the GL:water mixture the complete consumption of [PtCl_2_(en)] was observed after 24 h, while in the EG:water mixture, the unreacted complex was still detected by ^1^H-NMR at the same time. The K_2_PtCl_4_ dissolved in water afforded a red-orange solution when mixed in both selected DESs. As the reaction with *en* goes on, K_2_PtCl_4_ is consumed, and discolouration of the solution is more rapid (within 24 h) in the EG:water mixture. Concurrently, the solutions became transparent, with the disappearance of the [PtCl_2_(en)] peaks in the spectra and the unexpected precipitation of the purple Magnus-type salt [Pt(en)_2_][PtCl_4_] [23].

For both DES:water solutions in ^1^H NMR spectra H_w_ and H_y_ peaks were found deshielded with respect to the water signal, indicating the coordination of *en* to platinum [25] while -CH_2_-_en_, H_x_, and H_z_ peaks are shielded with respect to DESs signals. [^1^H,^13^C]-HSQC allowed us to resolve the overlapping signals belonging to H_x_ and -CH_2_-_en_. In ^195^Pt experiments, one signal for each of the reaction products was observed. Even though the ^195^Pt resonance window is very large (~15,000 ppm), NMR signals are very sensitive to the chemical environment; therefore, substituting even very similar ligands can change chemical shifts in the order of hundreds of ppm, allowing us to easily monitor reactions [28]. Among the factors that affect ^195^Pt chemical shifts, the solvent used in ^195^Pt-NMR spectroscopy can also significantly influence the δ_Pt_ value [35]. In this set of experiments, the δ values obtained from ^195^Pt experiments resulted very similar in both DES:water mixtures (Table 5). Given the significant dependence of ^195^Pt chemical shifts on the solvation environment [27,36], this suggests similarity in the solvation environment of K_2_PtCl_4_ and the products in the two DES:water solutions. These can be compared to the ^195^Pt δ obtained for the same species in traditional solvents, also reported in Table 5.

In both cases water content changed the viscosity of DESs, affecting the Pt(II) salt reactivity. In GL–water mixture we observed the quick formation of [PtCl_2_(en)] followed a few hours later by the formation of [Pt(en)_2_]Cl_2_. Twenty-four hours later we were not able to observe the [PtCl_2_(en)] peak in ^1^H and ^195^Pt spectra, due to the complete consumption of mono-addition product.

In EG–water mixture, after the rapid formation of [PtCl_2_(en)], we observed formation of [Pt(en)_2_]Cl_2_ in the following few hours. However, the consumption of [PtCl_2_(en)] resulted slower than in GL:water and peaks still resulted present in ^1^H and ^195^Pt spectra 24 h later.

In both DES:water solutions kept for 30 days at 7 °C, free ethylenediamine and [Pt(en)_2_]Cl_2_ could still be observed, while the mono-addition product signal was absent, due to its complete consumption. In the reactions carried out in both DES:water mixtures, [Pt(en)_2_]Cl_2_ further reacted with K_2_PtCl_4_ leading to the formation and precipitation of a purple Magnus-type salt, while *en* remained associated with DES. In the 1:1 Pt-to-*en* ratio reactions, the residual free *en* could be explained by its strong association with DESs, shown by DOSY experiments, making it unavailable for further reaction. The observed discolouration of the solution due to consumption of PtCl_4_^2^**^−^** could also be dependent on the partial inclusion of the anions within the precipitate.

Some differences also occurred in the formation of the Magnus-type salt. Due to the intrinsic higher viscosity of glyceline than ethaline DESs (η_GL_ = 199.00 mPa·s; η_EG_ = 30.90 mPa·s at 308.15 K for pure DES solution [37,38]), the bis-cationic product and residue K_2_PtCl_4_ had lower mobility, resulting in a slower formation of the purple Magnus-type salt, while in EG:water mixture the higher mobility of [Pt(en)_2_]Cl_2_ promoted slightly faster formation of Magnus salt. In *en* excess conditions for both DESs, we observed both the precipitation of Magnus salt and the precipitation of [Pt(en)_2_]Cl_2_ in the form of white needles, after following a long storage of the NMR tubes in the fridge at 7 °C.

Differences in diffusion coefficient (D) values obtained from NMR experiments depend on molecular sizes and shapes, temperature, and solvent viscosity, according to the Stokes–Einstein equation [39]. DOSY experiments gave comparable results. Diffusion coefficients obtained for water molecules in both DES:water solutions (D_wt-GL_ = 1.21 × 10^−10^ m^2^·s^−1^, D_wt-EG_ = 3.57 × 10^−10^ m^2^·s^−1^) were much lower than those measured in pure water (D_H2O_ = 2.19 × 10^−9^ m^2^·s^−1^) [33]. In both solutions, as expected based on molecular weight and size, the mobility of DES components resulted in much lower than water mobility. The free ethylenediamine and the platinum complexes had similar diffusion coefficient values, closer to those measured for the DES components than those of water. TSP resulted in both mixtures being the less mobile species. The two peaks of exchangeable H_w_ (-NH_2_ of [Pt(en)_2_]Cl_2_) and H_y_ (-NH_2_ of [Pt(en)_2_]Cl_2_) nuclei showed intermediate diffusion coefficients. Consistently with the lower viscosity of EG:water solution, all molecules had higher mobility in this mixture than in GL:water solution (Table 2 and Table 4). The water diffusion coefficient obtained in EG:water solution was approximately three times greater than those obtained in GL:water solution. Choline chloride mobility in EG:water mixture was nearly 2.5 times greater than the one observed in GL:water. In both solvents, choline chloride signals resulted closely associated with their respective hydrogen bond donors. Diffusion coefficients measured for free *en* and reaction products were nearly two times higher in the EG:water mixture.

For small rapidly tumbling molecules, faster motions correspond to slower T1 relaxation times [40]. The experiments performed showed comparable behaviour for both DES:water solutions. While water content reduces the viscosity of both DESs [19,41], the ternary mixtures maintain higher viscosity compared to pure water. Thus water molecules in both mixtures are more affected by the lattice and have a higher tumbling rate, consistently with T1 relaxation times (T1_wt-GL_ = 0.05 s, T1_wt-EG_ = 0.07 s) shorter than those measured in pure water (T1_wt_ = 4.96 s) [42].

Choline chloride methyl protons (H_d_ and H_d′_) in both DESs relaxed at a rate close to the one displayed by water molecules. As reported in Table 3 and Table 4, T1 values obtained for other DES signals, TSP, reaction products, and free *en* were generally shorter in the GL:water mixture than in the EG:water mixture. In both solutions, TSP showed a long relaxation time, due to more limited interactions with the environment, consistently with the low mobility observed in both GL:water and EG:water solutions.

## 3. Materials and Methods

### 3.1. Starting Materials

Ethylenediamine (*en*) was purchased from Alfa Aesar (Fisher Scientific Italia, Segrate (MI), Italy); Deuterated solvent D_2_O was purchased by Sigma-Aldrich (Merck Life Science S.r.l., Milano, Italy). Trimethylsilylpropanoic acid (TSP), used as nuclear magnetic resonance (NMR) internal standard, was purchased from Isotec (Merck Life Science S.r.l., Milano, Italy). Choline chloride, glycerol, and ethylene glycol were purchased from Sigma-Aldrich (Merck Life Science S.r.l., Milano, Italy). All reagents were used as purchased, without further purifications.

### 3.2. DESs Preparation

Deep Eutectic Solvents (DESs) used in the reactions were prepared according to previously reported procedures. The solvents used were glyceline (GL) formed by choline chloride and glycerol (ChCl:GLY, 1:2 ratio) and ethaline (EG) formed by choline chloride and ethylene glycol (ChCl:GC, 1:2 ratio) [43].

### 3.3. NMR Spectroscopy

NMR spectra were acquired on a Bruker Avance III 600 MHz equipped with a broadband observe probe and on a Bruker Avance III 400 MHz equipped with an inverse broadband probe (Bruker Italia S.r.l., Milano, Italy). Spectra were acquired for ^1^H, ^13^C, ^15^N, and ^195^Pt in natural abundance. ^1^H NMR spectra were acquired on a Bruker Avance III 600 MHz with a standard Bruker zg pulse sequence. A total of 16 scans were collected into 65 K data points with a relaxation delay of 2.0 s. A spectral width of 14 ppm and acquisition time of 5.8 s were selected.

^195^Pt NMR spectra were acquired on a Bruker Avance III 400 MHz, with a standard Bruker zgpg pulse sequence. A total of 49,152 scans were collected into 130 K data points with relaxation delay of 0.5 s. A spectral width of 2530 ppm and acquisition time of 0.3 s were selected.

[^1^H,^13^C]-HSQC spectra were acquired on a Bruker Avance III 600 MHz, with a standard Bruker pulse sequence (hsqcetgpsisp2.3) with 15 and 230 ppm spectral widths in the proton and carbon dimensions, respectively, 4 K data points in f2, and 128 increments in f1.

[^1^H,^15^N]-HSQC spectra were acquired on a Bruker Avance III 600 MHz, with a standard Bruker pulse sequence (hsqcetgpsisp2.3) with 13 and 250 ppm spectral widths in the proton and carbon dimensions, respectively, 2 K data points in f2, and 64 increments in f1.

^1^H and ^13^C chemical shifts (δ) were referenced to internal sodium trimethylsilylpropanoic acid (TSP). ^15^N spectra were referenced to external standard 1.5 M NH_4_Cl in 1 M HCl set at 23.6 ppm. ^195^Pt spectra were referenced to external H_2_PtCl_4_ in D_2_O at 0 ppm. NMR spectra were acquired in a 70:30% *v*/*v* mixture of the selected DES and water (1:1 H_2_O and D_2_O), added of a minimal amount of TSP for chemical shift reference. Diffusion experiments were performed on a Bruker Avance III 400 MHz, by using a stimulated echo sequence with bipolar gradient pulses (Bruker pulse program *ledbpgp2s* [44]) with a spectral width of 12 ppm centred at 5 ppm, and 16 K data points. *Δ* was set to 200 ms and δ to 2.5 µs. Gradient pulses strength was varied over 16 steps to obtain a 95% decrease in the resonance intensity at the largest gradient amplitudes. The self-diffusion coefficients D, were calculated by fitting the intensity of each signal to the exponential function:

$$I_{\left(g\right)}=I_{0}\exp\left[-D\gamma H^{2}g^{2}\delta^{2}\left(\frac{\Delta -\delta }{3}\right)\right]$$

where *I(g)* is the resonance intensity measured for a given gradient strength, *g*; *I_0_* is the NMR signal in the absence of the gradient pulse; *γH* is the gyromagnetic ratio of the hydrogen nucleus; *δ* (little delta) is the duration of the bipolar gradient pulse; and *Δ* (big delta) is the observation time.

Spin–Lattice (T1) NMR relaxation time experiments were performed on a Bruker Avance III 400 MHz. T1 were measured using inversion recovery experiments (Bruker pulse program T1ir [40]), the relaxation delay D1 was 12 s for the ^1^H experiments, and the VD list contained sixteen delays (0.00001–6 S); T1 were calculated by fitting the intensity of each signal to the exponential function:

$$M_{z}\left(t\right)=M_{0}\left(1-e^{-t∕T_{1}}\right)$$

where *M_z_(t)* is magnetization along the *z*-axis at time t; *M_0_* is Equilibrium magnetization; and T1 is time constant for longitudinal relaxation.

NMR spectra were processed with Topspin 3.6.5, and DOSY and T1 experiments fitting were performed with Dynamics centre 2.8.5 (Bruker Italia S.r.l., Milano, Italy).

## 4. Reactions

### 4.1. Synthesis of K_2_PtCl_4_

The synthesis of K_2_PtCl_4_ was carried out according to the methods previously reported in the literature with slight modifications [45]. Briefly, 56.38 mmol of Pt wire (0.25 mm diameter), cut into pieces of ~1 cm length, are placed in 120 mL of *aqua regia* (HCl:HNO_3_ 75:25%, *v/v*) and heated until dissolution. During the reaction, the formation of red-orange NO_2_ gas is observed. Once the volume is halved, increasingly diluted HCl solutions (12 to 4 M) are added to obtain H_2_PtCl_6_, until the NO_2_ is no longer observed. The solution is then allowed to cool. A total of 112.77 mmol of KCl are dissolved in the minimum amount of hot water and added to the previous solution containing 56.38 mmol of H_2_PtCl_6_. K_2_PtCl_6_ immediately forms as a yellow precipitate, which is recovered by filtration, washed with cold H_2_O, 99.9% EtOH, and Et_2_O, and then dried with dry air (55.79 mmol, yield 98.85%). This operation is repeated multiple times to precipitate all the K_2_PtCl_6_, which is then suspended in 300 mL of distilled water. To the K_2_PtCl_6_ suspension, 27.89 mmol of N_2_H_4_·2HCl are added: the first half is added at room temperatures, and the mixture is stirred for 2 h. The second half is added after dissolution in 15 mL of distilled water and slowly dripped into the suspension containing K_2_PtCl_6_ and the first portion of N_2_H_4_·2HCl, with the system thermostated at 50 °C. The reaction mixture is stirred for 4 h at 50 °C and then for 2 h at 60 °C, during which a colour change of the solution from yellow to burgundy red is observed, indicating the formation of soluble K_2_PtCl_4_. The aqueous K_2_PtCl_4_ solution is evaporated to dryness under reduced pressure at 50 °C, yielding a burgundy-red solid that is re-dissolved in ice cold water and filtered. The filtrate is evaporated to dryness, and the solid K_2_PtCl_4_ obtained is washed with acetone, MeOH, and Et_2_O and dried under vacuum, yielding 54 mmol (94% yield) [46].

### 4.2. Synthesis of [Pt(en)Cl_2_] (En = Ethylenediamine)

[Pt(en)Cl_2_] was synthesised in cold water, following the method of Rochon et al. [21].

### 4.3. Reaction of K_2_PtCl_4_ and Ethylenediamine in DES

Reactions of K_2_PtCl_4_ and *en* were performed in the 70:30% *v*/*v* DES:water mixtures. In order to dissolve K_2_PtCl_4_ and obtain deuterium lock for NMR analysis, the platinum salt was previously dissolved in a measured amount of D_2_O.

For the 1:1 reaction stoichiometry, 150 µL of a 0.05 mmol D_2_O solution of K_2_PtCl_4_ were added to 700 µL of GL or EG DESs and successively mixed with 150 µL of a 0.05 mmol ethylenediamine (*en*) in H_2_O solution. The resulting solution was stirred in the dark at room temperature.

Analogously, for the 1:2 stoichiometry, 150 µL of a 0.05 mmol D_2_O solution of K_2_PtCl_4_ were added to 700 µL of GL or EG DESs and successively mixed with 150 µL of a 0.10 mmol *en* in H_2_O solution. The resulting solution was stirred in the dark at room temperature. All the solutions were transferred into an NMR tube for performing NMR analyses.

### 4.4. Preparation of Standard Ethylenediamine in DES:Water Solutions

150 µL of an H_2_O solution 0.05 mmol of *en*, 150 µL of D_2_O and 700 µL of GL, and EG DESs were stirred together in the dark at room temperature.

## 5. Conclusions

In this work, the model reaction between K_2_PtCl_4_ and ethylenediamine (*en*) was studied in mixtures composed of hydrophilic deep eutectic solvents choline chloride-glycerol 1:2 (GL) or choline chloride-ethylene glycol (EG), and water (1:1 H_2_O:D_2_O) 70:30% *v*/*v*.

In both solvent mixtures, the reaction with *en* led to the quick formation of [PtCl_2_(en)], followed by further reaction of an additional *en* moiety with the previously formed [PtCl_2_(en)] complex, affording [Pt(en)_2_]Cl_2_. Synthesized compounds were characterised in DES–water mixtures by ^1^H, ^195^Pt NMR spectra and by 2D [^1^H,^13^C]-HSQC and [^1^H,^15^N]-HSQC NMR experiments. In all solvents and at different Pt-to-*en* ratios (1:1 and 1:2) we observed a progressive discolouration of the solution due to the consumption of K_2_PtCl_4_. Additionally, for longer times, the consumption of the mono-addiction product, confirmed by NMR spectra, led to the unexpected precipitation of the purple Magnus-type salt [Pt(en)_2_][PtCl_4_]_2_. While the consumption of [PtCl_2_(en)] resulted slower in the EG:Water mixture, the formation of Magnus-type salts and discolouration of the solution resulted slightly slower in the GL:water mixture, indicating that different composition of DESs was able to affect differently the metallic centre reactivity and the environment leading to the precipitation.

To explore this unexpected behaviour, we analysed the solution using diffusion-ordered spectroscopy (DOSY) and T_1_ relaxation experiments on ^1^H nuclei. The results were comparable for both solvents: water mobility was lower than in pure water, the mobility of DES components was significantly lower than that of water in the mixture, while the diffusion coefficients of the non-labile [PtCl_2_(en)], [Pt(en)_2_]Cl_2_, and residual *en* protons were comparable to each other and to those of the DES, indicating that the reaction products are associated with DES molecules. However, the labile -NH_2_ protons diffusion coefficients showed a different pattern, having higher values, similar to those observed for the water molecules, suggesting that water is able to travel rather efficiently among DES molecules and provide fast hydrogen exchange.

Further investigations are warranted to improve the understanding of the reactivity of platinum(II) complexes in DESs with the perspective of developing more effective and sustainable one-step synthetic pathways for therapeutically improved cisplatin analogues in order to avoid silver halides typically derived from traditional Pt(II) complexes synthesis. While the results presented here show that the choline chloride-polyol type DESs employed afford unwanted side reactions in the model reaction studied, further investigations in other DES types, including lipophilic ones, could allow us to attain previously unavailable reaction pathways.

## Figures and Tables

**Table 1:** Chemical shift (δ, ppm) values for free ethylenediamine and reaction products in GL:water 70:30% *v*/*v* mixture, obtained from [^1^H,^13^C]-HSQC and [^1^H,^15^N]-HSQC NMR experiments.

	^1^H	^13^C	^15^N
-CH_2_-_en_	2.67	45.90	-
H_x_	2.66	49.14	-
H_y_	5.34	-	−31.37
H_z_	2.58	51.21	-
H_w_	5.31	-	−31.42

**Table 2:** Diffusion coefficient and T1 relaxation time values obtained in GL:water 70:30% (*v*/*v*) mixture. *n.d.* signal to noise ratio too low for the fitting.

GL	δ (ppm)	D (10^−10^ m^2^/s)	D Error (10^−10^ m^2^/s) ^1^	T_1_ (*s*)	T_1_ Error ^1^
H_b_	4.03	0.49	1.01 × 10^−5^	0.59	1.81 × 10^−5^
H_c_	3.55	0.50	4.50 × 10^−5^	0.34	5.74 × 10^−6^
H_d_	3.22	0.47	4.40 × 10^−6^	0.10	4.21× 10^−7^
H_f_	3.59	0.56	2.27 × 10^−5^	0.32	6.07 × 10^−6^
H_g_	3.72	0.55	7.82 × 10^−6^	0.66	1.91 × 10^−5^
water	4.81	1.21	1.29 × 10^−5^	0.05	1.65 × 10^−7^
H_x_	2.65	0.42	1.72 × 10^−3^	0.29	3.31 × 10^−3^
H_y_	5.34	1.11	3.18 × 10^−2^	0.26	1.00 × 10^−3^
H_z_	2.57	0.45	6.65 × 10^−3^	0.22	1.14 × 10^−2^
H_w_	5.30	1.03	2.52 × 10^−2^	*n.d.*	*n.d.*
-CH_2_-en	2.67	0.52	4.66 × 10^−4^	0.49	1.80 × 10^−4^
TSP	0.00	0.33	7.29 × 10^−5^	0.95	7.20 × 10^−4^

^1^ Fit parameter Error estimation method: from Monte Carlo simulations.

**Table 3:** Chemical shift values (ppm) for free *en* and reaction products in EG:water 70:30% *v*/*v* mixture obtained from [^1^H,^13^C]-HSQC and [^1^H,^15^N]-HSQC NMR experiments.

	^1^H	^13^C	^15^N
-CH_2_-_en_	2.69	45.44	-
H_x_	2.68	49.53	-
H_y_	5.42	-	−30.93
H_z_	2.58	51.76	-
H_w_	5.35	-	−30.44

**Table 4:** Diffusion coefficient and T1 relaxation time values obtained in EG:water 70:30% *v*/*v* mixture. *n.d.* signal to noise ratio too low for the fitting.

EG	δ (ppm)	D (10^−10^ m^2^/s)	D Error (10^−10^ m^2^/s) ^1^	T_1_ (s)	T_1_ Error ^1^
H_b′_	4.03	1.26	6.04 × 10^−4^	0.94	2.94 × 10^−5^
H_c′_	3.55	1.27	2.62 × 10^−5^	0.80	2.16 × 10^−5^
H_d′_	3.25	1.24	2.55 × 10^−5^	0.07	3.11 × 10^−7^
H_h′_	3.63	1.78	3.52 × 10^−5^	0.08	3.70 × 10^−7^
water	4.78	3.57	1.01 × 10^−4^	0.07	3.28 × 10^−7^
H_x_	2.65	1.04	1.91 × 10^−2^	0.34	9.45 × 10^−3^
H_y_	5.34	1.78	1.30 × 10^1^	0.46	3.66 × 10^−3^
H_z_	2.57	0.99	3.94 × 10^−2^	0.39	3.43 × 10^−4^
H_w_	5.30	2.23	7.65 × 10^−2^	*n.d.*	*n.d.*
-CH_2_-_en_	2.67	1.41	1.57 × 10^−2^	0.57	1.40 × 10^−3^
TSP	0.00	0.87	1.42 × 10^−3^	1.41	1.34 × 10^−3^

^1^ Fit parameter Error estimation method from Monte Carlo simulations.

**Table 5:** ^195^Pt NMR chemical shifts for the complexes in DES and traditional solvents. [PtCl_2_(en)] NMR data are reported for DMSO solution since the complex is insoluble in water.

	(δ) ppm (GL)	(δ) ppm (EG)	(δ) ppm	Ref.
K_2_PtCl_4_	−1560	−1561	−1620 (in D_2_O)	[28]
[PtCl_2_(en)]	−2384	−2386	−2345 (in DMSO)	[29]
[Pt(en)_2_]Cl_2_	−3030	−3030	−3015 (In D_2_O)	[26]

## Data Availability

Data available upon request.

## Supplementary Materials

The following supporting information can be downloaded at: https://www.mdpi.com/article/10.3390/molecules30091890/s1, Figure S1: stacked ^1^H NMR spectra of Reaction 1 in the GL:water mixture 70:30% *v*/*v*; Figure S2: stacked ^1^H NMR spectra of Reaction 2 in the EG:water mixture 70:30% *v*/*v*; Figure S3: Expansions of [^1^H,^13^C]-HSQC NMR spectra acquired 30 days later.
