# Supplementary material for: Reactive Behaviour of Platinum(II) Salts with Ethylenediamine in Sustainable Water/Choline Chloride-Based Deep Eutectic Solvents Mixtures
Source: Molecules. 2025 Apr 24;30(9):1890. doi: 10.3390/molecules30091890 (PMC12073102; doi:10.3390/molecules30091890)
Supplement: Supplementary file 1 [file molecules-30-01890-s001.zip › molecules-3582722-supplementary.pdf]

molecules

Supplementary materials

Reactive Behaviour of Platinum(II) Salts with Ethylenediamine in Sustainable Water/Choline Chloride-Based Deep Eutectic Solvents Mixtures

Nicola Garofalo [1], Francesco Messa [1], Alessandra Barbanente [2], Francesco Paolo Fanizzi [1], Antonio Salomone [2], Nicola Margiotta [2] and Paride Papadia [1,*]

[1] Department of Biological and Environmental Sciences and Technologies (DiSTeBA), University of Salento, 73100 Lecce, Italy; nicola.garofalo@unisalento.it (N.G.); francesco.messa@unisalento.it (F.M.); fp.fanizzi@unisalento.it (F.P.F.)

[2] Dipartimento di Chimica, Università degli Studi di Bari Aldo Moro, Via E. Orabona 4, 70125 Bari, Italy; alessandrabarbanente@libero.it (A.B.); antonio.salomone@uniba.it (A.S.); nicola.margiotta@uniba.it (N.M.)

* Correspondence: paride.papadia@unisalento.it

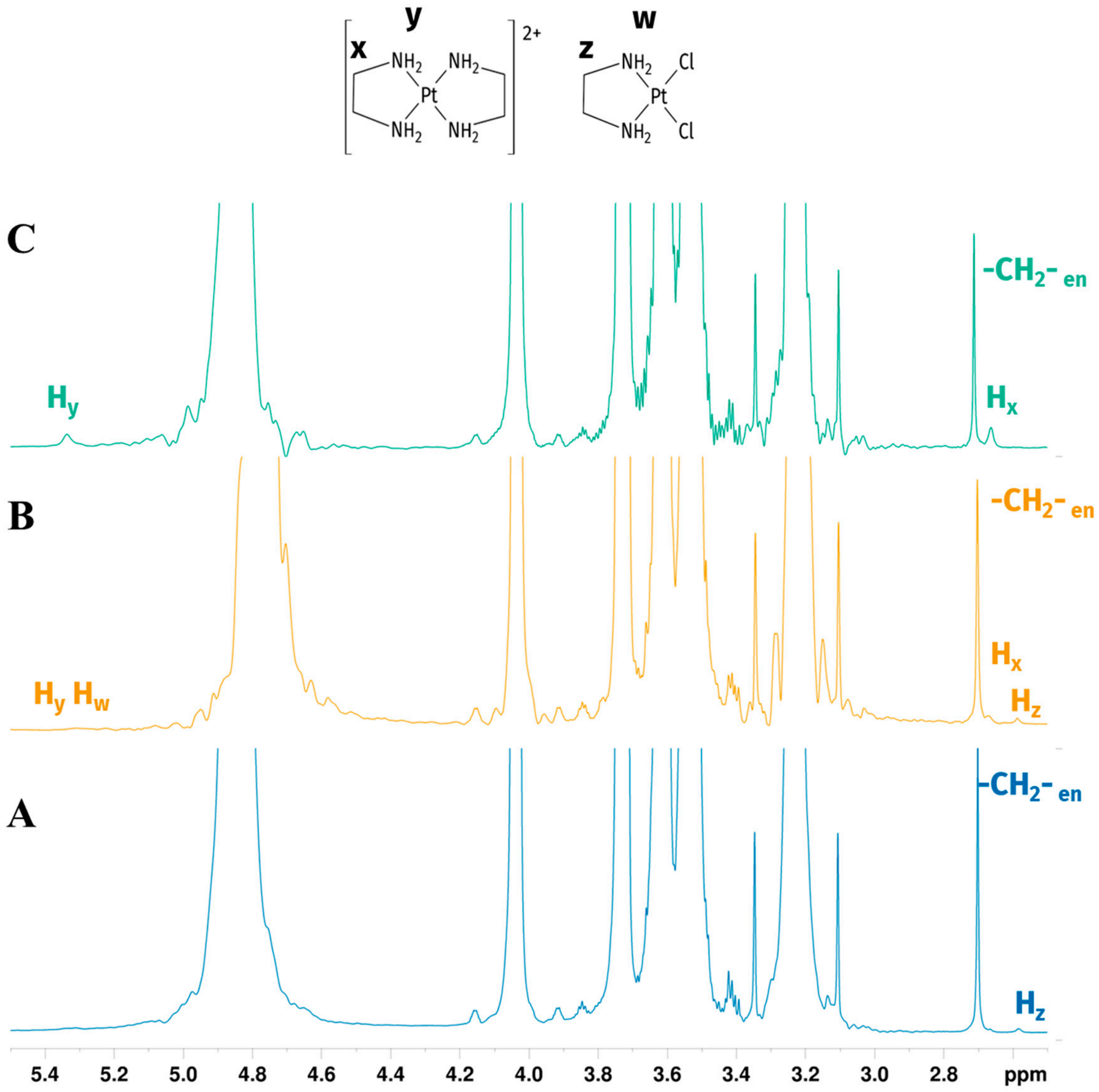

Figure S1: stacked ^{1}H NMR spectra of reaction 1 in the GL:water mixture 70:30% v/v: **A**) showing the early formation of mono-addiction product at the beginning of reaction; **B**), indicating the reaction goes on, reaching the formation of biscathionic product after four hours; **C**) showing the consumption of $[PtCl_2(en)]$ 24 hours later.

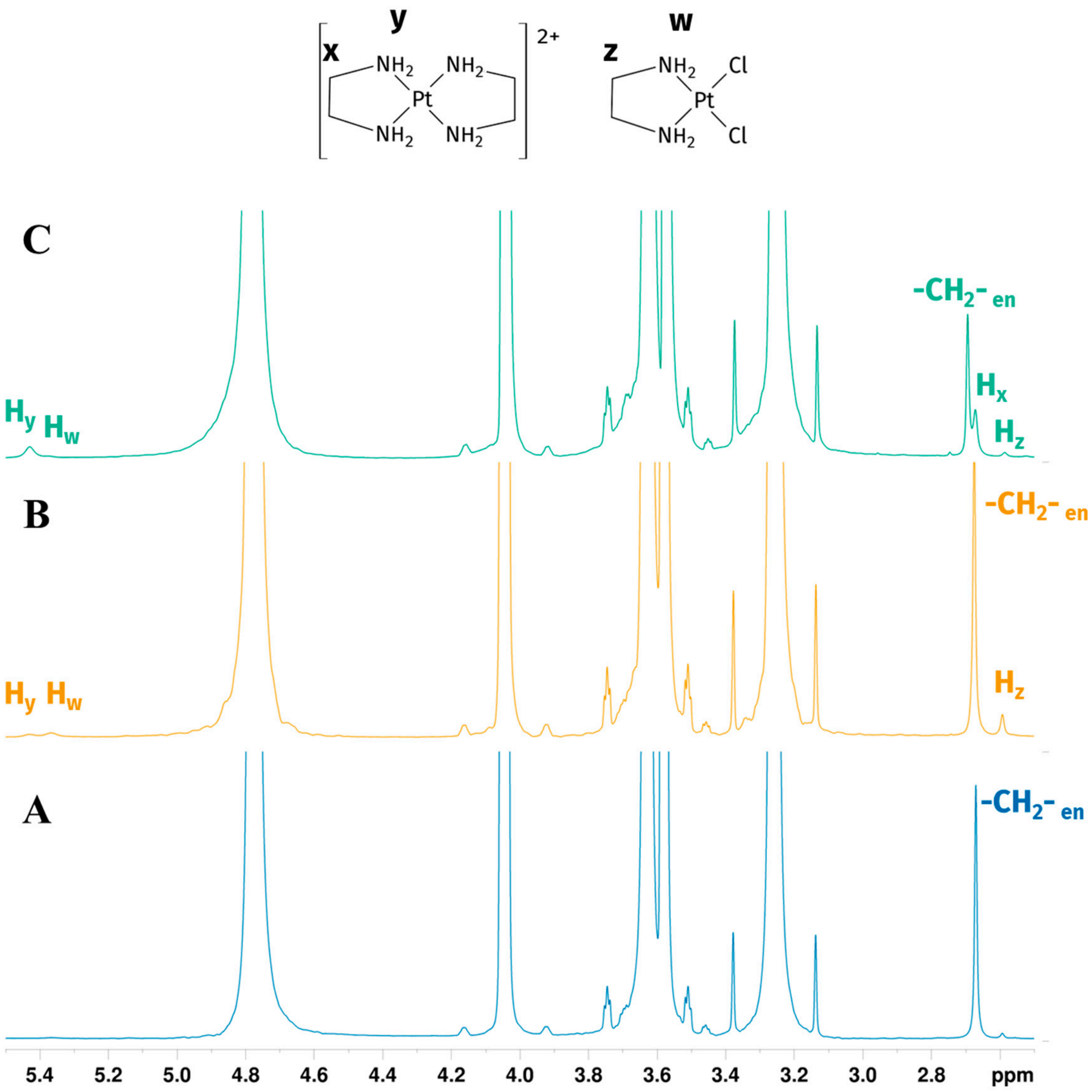

Figure S2: stacked ^{1}H NMR spectra of reaction 2 in the EG:water mixture 70:30% v/v: **A**) showing the early formation of mono-addiction product at the beginning of reaction; **B**), indicating the reaction goes on, reaching the formation of biscathionic product after one hour; **C**) showing the partial consumption of $[PtCl_2(en)]$ 24 hours later.

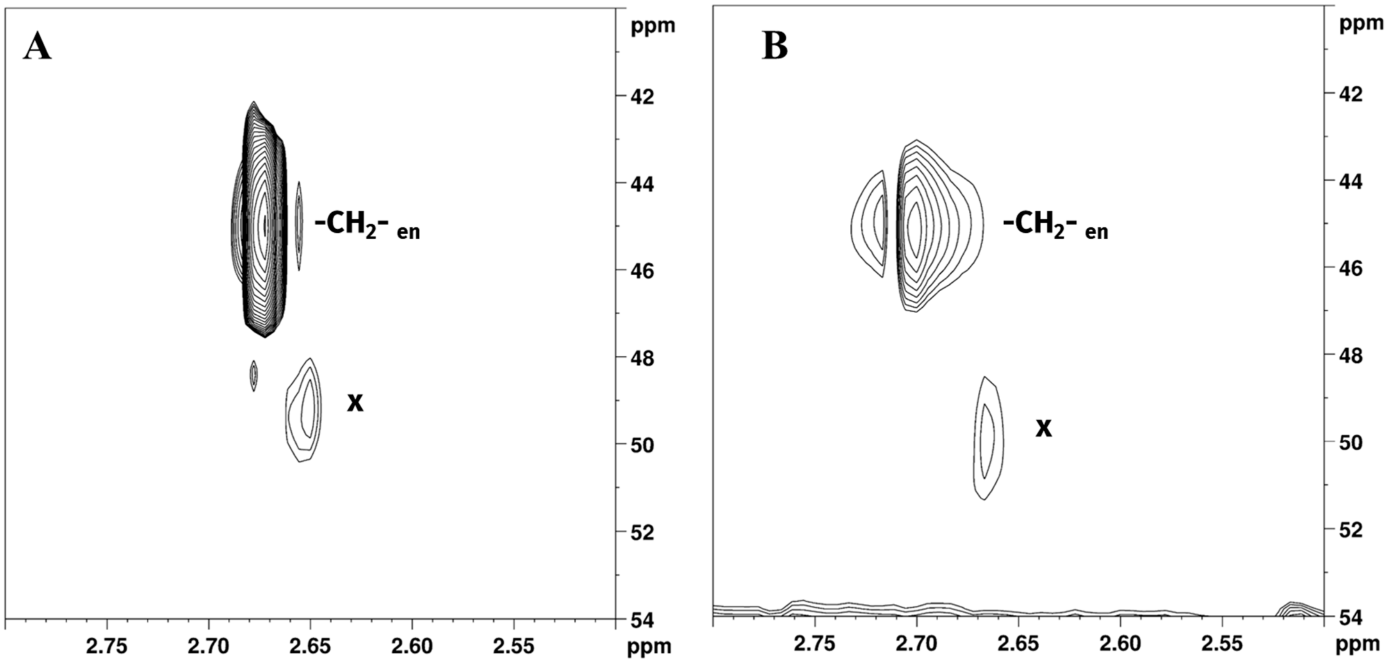

Figure S3: Expansions of [^1H,^{13}C]-HSQC NMR spectra acquired 30 days later, in which are still detectable peaks belonging to -CH_{2}-$_{en}$ (free *en*) and x (-CH_2- of $[Pt(en)_2]^{2+}$) in **A**) GL:water 70:30% v/v mixture and **B**) EG:water 70:30% v/v mixture.
